# Endoscopic View of the Aorta Through the Oesophagus: An Unusual Complication Post Thoracic Endovascular Aortic Repair

**DOI:** 10.1155/crgm/6277906

**Published:** 2025-01-10

**Authors:** Kushedison Yunus, Hasib Ahmadzai, Eleanor Noreen Dunlop, Andrew Thomson

**Affiliations:** Gastroenterology and Hepatology Unit, The Canberra Hospital, Australian Capital Territory, Canberra, Australia

## Abstract

We present a case of an 80-year-old female who presented with chest pain, vomiting and night sweats a few weeks post thoracic endovascular aortic aneurysm repair (TEVAR). A computed tomography (CT) scan demonstrated a type 1B endoleak for which she underwent a repeat TEVAR. Postoperatively, she developed fever, dysphagia, haematemesis and melaena. CT angiography subsequently confirmed an aorto-oesophageal fistula (AEF). Gastroscopy was performed to confirm this and found an ovoid oesophageal perforation with visible aortic graft and purulent fluid. Serial endoscopic oesophageal stents were placed and the patient recovered after an oesophageal Ultraflex stent was placed. Unfortunately, however, the patient was found unresponsive at home with black vomitus and in cardiac arrest and passed away 18 months after her initial endoscopic procedure. This case highlights that AEF is a complication following a TEVAR procedure. This can be managed temporarily with oesophageal stent placement and an Ultraflex stent in the longer term. However, oesophageal stent placement is not curative in cases of significant oesophageal perforation as it does not lead to lead to closure of a large defect.

## 1. Main Manuscript

**Figure 1:**
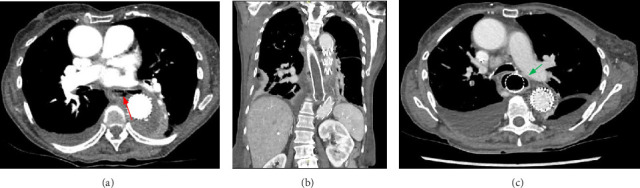
(a) Computed tomography (CT) images showing aorto-oesophageal fistula post repeat TEVAR (red arrow). (b-c) Images showing persisting oesophageal defect after oesophageal stent insertion (green arrow).

**Figure 2:**
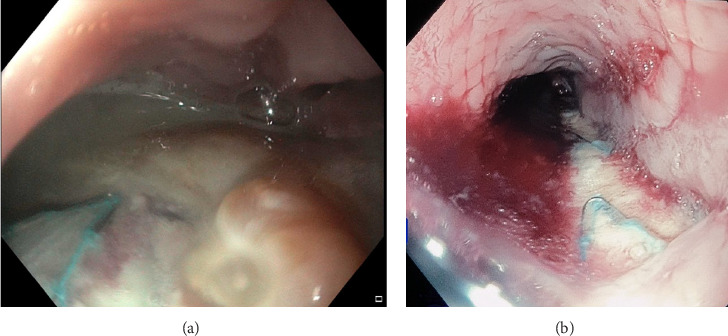
(a) Initial endoscopic view of aorto-oesophageal defect with purulent material seen in the mediastinum. (b) Repeat gastroscopy 10 months later showing persisting defect with aortic graft visible through the oesophagus with subsequent Ultraflex stent insertion.

An 80-year-old female, who had undergone a thoracic endovascular aortic repair (TEVAR) for idiopathic aortic arch perforation four weeks previously, presented with acute chest pain, vomiting and night sweats. Computed tomography (CT) angiography demonstrated a type 1B endoleak, for which a repeat TEVAR was performed to cover a leak through the existing graft. Postoperatively, she developed fever, dysphagia, haematemesis and melaena. Repeat CT angiography ruled out bleeding into the mediastinum but demonstrated a communication between the aorta and oesophagus, suggestive of an aorto-oesophageal fistula (AEF) (Figure 1). Gastroscopy confirmed this diagnosis, with a 3 cm ovoid oesophageal perforation with visible aortic graft and purulent fluid 30 cm from the incisors (Figure 2), which was managed with a partially covered 12.5 cm × 25 mm oesophageal stent. Endoscopic biopsies showed nonmalignant ulcerated tissue. Given the purulent fluid in the oesophagus there were concerns for medastinitis, so she was managed with long-term antibiotics. She recovered from this admission and was discharged home 2 weeks later, on long-term amoxicillin and feeding through a percutaneous jejunostomy (PEJ) tube. With subsequent placement of a fully covered oesophageal stent over the previous partially covered stent, she was able to tolerate oral intake. A series of oesophageal stenting procedures were then performed with both partially covered and fully covered stents over the following months to allow healing of the defect; however, the oesophageal defect did not recover. The oesophageal stents thus remained in situ over the following 6 months. She did report occasional episodes of postprandial coughing and retching on follow-up outpatient clinic reviews.

Six months after the first endoscopic procedure, she presented to the emergency department with haemoptysis and melaena. CT angiography found a new thrombus within the TEVAR graft adjacent to the oesophageal stent and an enlarged oesophageal defect with adjacent pulmonary consolidation. Just prior to an emergency gastroscopy, she developed haematemesis and a large blood clot was found within the oesophagus. A third TEVAR graft was then urgently placed within the pre-existing aortic stents. Despite a further fully covered oesophageal stent insertion, subsequent Barium swallow now revealed fluid extravasation outside the stent into the mediastinum. Given the patient's age and frailty, she was deemed not to be a candidate for open thoracic surgery to repair the oesophagus. During this admission, she remained on long-term intravenous antibiotics to treat pneumonia and mediastinitis. The oesophageal defect was subsequently managed 10 months after the initial procedure with an oesophageal 23 × 120 mm covered Ultraflex stent (Boston Scientific, USA), which led to symptomatic improvement with no leakage seen at subsequent Barium swallow. She underwent rehabilitation for physical deconditioning and was tolerating solid food on discharge.

Unfortunately, however, 18 months following her initial endoscopic procedure, she was found unresponsive at home with black vomitus and in cardiac arrest. She was unable to be revived and passed away shortly thereafter.

Although infrequent, AEF is a complication of TEVAR, which if untreated is fatal [1]. Fistula formation between the aorta and the oesophagus may arise from direct pressure necrosis or ischaemia of the oesophageal wall adjacent to the aortic stent [1]. A contrast-enhanced CT-scan is valuable for evaluation and gastroscopy is required for diagnostic confirmation. Placement of an oesophageal stent is not curative to the extent that it has not been shown to lead to closure of the oesophageal perforation [2]. In comparison, radical surgery such as oesophagectomy with extensive aortic reconstruction is the only proven curative strategy [2, 3]. This case highlights that placement of a partially covered Ultraflex stent may be the optimal management in patients not fit for surgery.

## Data Availability

The data that support the findings of this study are available on request from the corresponding author (Andrew Thomson).
